# Looking back and abroad while (not) moving forward. Migration, ideas and the stability of citizenship in Spain

**DOI:** 10.3389/fsoc.2025.1570110

**Published:** 2025-07-03

**Authors:** Francesco Pasetti, Reinhard Schweitzer

**Affiliations:** ^1^CIDOB—Barcelona Center for International Affairs, Barcelona, Spain; ^2^Department of Law and Political Science, Universitat Abat Oliba CEU, CEU Universities, Barcelona, Spain

**Keywords:** citizenship law, politics, Spain, ideas, frames, migration-citizenship nexus, policy

## Abstract

This article addresses the remarkable stability of the Spanish citizenship regime. Since it was established in 1982, it has remained largely unchanged, despite the country's rapid transformation from a country of emigration to a major destination for non-EU immigrants. We complement existing explanations for this phenomenon by shifting the analytical focus to the realm of ideas. Based on a close analysis of the law-making process and parliamentary debates about citizenship reforms between 1978 and 2024, we argue that this puzzling stability can partly be attributed to the widely shared and remarkably stable way in which the country's political elite conceives nationality. We identify three constitutive elements that make this dominant citizenship frame: (i) the preference for blood-ties over territorial presence, (ii) the preferential treatment of emigrants (and their descendants) over immigrants, and (iii) the predilection for potential citizens' historical over contemporary connections to Spain. This set of ideas, in which political parties' views overlap, has constituted the tracks along which the country's nationality laws have evolved. It has outlived not only demographic but also political changes including the appearance of the country's first far-right, anti-immigrant party. By focusing on ideas, this article offers a new analytical and less deterministic perspective, complementing the explanatory backdrop provided to date by the scholarship concerned with citizenship law-making. Our findings and analysis contribute to a fuller understanding of the politics of citizenship in Spain and—more generally—of the ambiguous role that past, present, and future migratory dynamics (can) play in shaping—the evolution of citizenship law-making. It thereby also contributes to the literature on the multifaceted nexus between citizenship and migration and to broader debates on the importance of ideas in public policymaking.

## Introduction

Spain's migration history is unique in that the country's transformation from being a major country of emigration into one strongly characterized by immigration happened over the course of only a few decades.^1^ Between the mid-eighteenth century and the 1970s, several millions of Spaniards left the country, first following the colonial route to Latin America and later, since the 1960s, also toward European destinations. It was only during the 1990s, that Spain's net migration rate turned positive for the first time after immigration had started to grow gradually during the 1980s, and rapidly after the mid-1990s, fueled in particular by the arrival of newcomers from non-EU countries. In the first decade of the twenty-fist century the country experienced an unprecedented rise in the share of foreign population, which grew from around 2% in 2000 to over 12% in 2010. Although the net migration rate has since then not always been positive—e.g., during the years following the 2008 financial crisis or those marked by the COVID-19 lockdowns—the country has quickly become, and remains until today, one of Europe's top immigration destinations. As González Enríquez (2024) recently pointed out, the significant growth of the Spanish population—by 20% between 2000 and 2024—was entirely due to immigration.

Increasingly in the limelight of public and political concern, immigration became the target of an intense proliferation of policy measures (Zapata-Barrero et al., 2008). According to the DEMIG (2015) database, from 2000 to 2013 Spanish legislators have put into effect 104 immigration-related measures, covering a widening spectrum of policy areas. The vast majority of these changes affected migrants' access to residence permits, education, or the labor market, whereas only four of them were related to citizenship^2^ and all four extended citizenship rights for the Spanish diaspora (see Table 1). In this article, we conceive “citizenship” as synonymous with “nationality” and thereby follow Bauböck's (2010) understanding of the concept as primarily referring to a legal status (connected to certain rights and obligations) that links a State and its citizens.^3^ In terms of policy/making, we thus focus on regulations regarding the acquisition, transmission, and loss of nationality, as well as the degree of its exclusivity.

**Table 1:** Proposed changes to the Spanish citizenship regime 1981–2024.

**Year**	**Incumbent (main party of ruling majority)**	**Proponent (political orientation)**	**Title of the iniciative**	**Ref no**.	**Key content**	**Result**	**Notes**
1981	Center-right Government (UCD)	UCD (center-right)	Bill. Reform of Articles 17–26 of the Civil Code (Nationality)	121/000165	Setting out the principles of the Spanish Citizeship Regime, incl. modes of acquisition and loss, residence requirements, priviledged groups, etc.	Approved in Law 51/1982	The content of the proposal (121/000165) is substantially confirmed.
1990	Center-left Government (PSOE)	PSOE (center-left)	Bill Proposal on the Reform of the Civil Code Regarding Nationality.	122/000009	Easier acquistion and recovery and stronger protection against loss for emigrants (and their descendants) Quicker residence-based naturalization for refugees	Approved in Law 18/1990	The content of the proposal (122/000009) is substantially confirmed.
1995	Center-left Government (PSOE)	PP (center-right)	Bill Proposal to Amend the Civil Code Regarding the Recovery of Nationality	121/000080	Favoring nationality recovery for emigrants (and descendants) willing to return	Approved in Law 29/1995	The content of the proposal (121/000080) is substantially confirmed.
1996	Center-right Government (PP)	IU (left-wing)	Bill Proposal to Amend the Civil Code Regarding Nationality.	122/000018	Extending *jus soli* and liberalizing residence-based naturalization	Rejected	
1996	Center-right Government (PP)	PSOE (center-left)	Bill Proposal to Amend the Civil Code Regarding Nationality.	122/000048	Extending *jus soli* and liberalizing residence-based naturalization	Rejected	
1999	Center-right Government (PP)	IU (left-wing)	Bill Proposal to Amend the Civil Code Regarding the Acquisition and Recovery of Nationality	122/000232	Extending *jus soli* and liberalizing residence-based naturalization	Rejected	
1999	Center-right Government (PP)	PSOE (center-left)	Bill Proposal to Amend the Civil Code Regarding Nationality.	122/000246	Extending *jus soli* and liberalizing residence-based naturalization	Rejected	
2001	Center-right Government (PP)	PSOE (center-left)	Bill Proposal to Amend the Civil Code Regarding Nationality.	122/000102	Extending *jus soli* and liberalizing residence-based naturalization	Submitted to initiative 122/000216	In the follow-up initiative (122/000216), the content of this proposal is discarded
2001	Center-right Government (PP)	PP (center-right)	Bill Proposal to Amend the Civil Code Regarding Nationality.	122/000109	Favoring nationality acquisition and recovery for Spaniards' descendants in Spain and abroad	Submitted to initiative 122/000216	In the follow-up initiative (122/000216), the content of this proposal is approved
2001	Center-right Government (PP)	IU (left-wing)	Bill Proposal to Amend the Civil Code Regarding Nationality.	122/000150	Extending *jus soli* and liberalizing residence-based naturalization	Submitted to initiative 122/000216	In the follow-up initiative (122/000216), the content of this proposal is discarded
2002	Center-right Government (PP)	PP (center-right)	Bill Proposal to Amend the Civil Code Regarding Nationality.	122/000216	Favoring nationality acquisition and recovery for Spaniards' descendants in Spain and abroad Re-enforcing the obligation to renounce original citizenship upon naturalization	Approved in Law 36/2002	The content of the proposal (122/000216) is substantially confirmed.
2006	Center-left Government (PSOE)	PSOE (center-left)	Bill on the Statute of Spanish Citizenship Abroad	121/000075	Ensuring equal social, economic, and political rights of Spanish citizens residing abroad	Approved in Law 40/2006	The content of the proposal (121/000075) is substantially confirmed.
2006	Center-left Government (PSOE)	PSOE (center-left)	Bill to Recognize and Expand Rights and Establish Measures in Favor of Those Who Suffered Persecution or Violence During the Civil War and the Dictatorship	121/000099	Favoring recovery of nationality for descendants of Spanish exiles (who emigrated due to civil war or dictatorship)	Approved in Law 52/2007	The content of the proposal (121/000099) is substantially confirmed.
2011	Center-left Government (PSOE)	PSOE (center-left)	Bill on the Civil Registry	121/000090	Extending nationality by option for descendants of exiled Spanish women	Approved in Law 20/2011	The content of the proposal (121/000090) is substantially confirmed.
2014	Center-right Government (PP)	PP (center-right)	Bill on the granting of Spanish nationality to Sephardic Jews originally from Spain.	121/000099	Facilitating naturalization path for Sephardic Jews	Approved in Law 12/2015	The content of the proposal (121/000099) is substatially confirmed.
2015	Center-right Government (PP)	PP (center-right)	Bill on administrative reform measures in the field of the Administration of Justice and the Civil Registry.	121/000101	Introducing civic and language tests for naturalization	Approved in Law 19/2015	The content of the proposal (121/0000101) is substantially confirmed.
2021	Center-left Government (PSOE)	PSOE (center-left)	Bill on Democratic Memory.	121/000064	Favoring recovery of nationality for Spaniards' descendants emigrated due to civil war or dictatorship (“extention” of the scope of Law 52/2007)	Approved in Law 20/2022	The content of the proposal (121/0000101) is substantially confirmed.
2021	Center-left Government (PSOE)	VOX (far-right)	Bill on the modification of the legal regime of nationality.	122/000176	Restricting requirements for acquisition through residence, option, and naturalization, and providing for the revocation in case of conviction for serious crimes against the State	Rejected	
2024	Center-left Government (PSOE)	VOX (far-right)	Organic Bill on the modification of the legal regime of nationality.	122/000054	Restricting requirements for acquisition, increasing the residency period, introducing language and knowledge requirements of Spanish history and culture, and providing for the loss of citizenship for certain crime	Rejected	

Selection: The debates and measures analyzed includes initiatives with “legislative function” issued in the Congress both by parties in government and in opposition, excluding those that have “expired” (i.e., not examined within the required legislative timeframe) and those “withdrawn” by the proponents themselves. In short, all legislative initiative that have been voted in Congress.

The Spanish citizenship regime has remained almost untouched since it was first established through Law 51/1982 and thus represents an area of legislation that is marked by an extraordinary degree of stability, as Martín-Pérez and Moreno-Fuentes (2012) have pointed out. Since the very beginning, this regime has been strongly anchored in the country's emigration and colonial histories (Rubio Marín et al., 2015), keeping *jus sanguinis* as the core principle ruling the acquisition and transmission of nationality,^4^ and granting preferential citizenship access to emigrants and their descendants, citizens of the former colonies, as well as Sephardic Jews. For example, all three groups are exempt from the general prohibition of dual nationality, the descendants of emigrants can easily recover Spanish nationality, and people from former colonies as well as Sephardic Jews can naturalize after (lawfully) residing in Spain for only 2 years, whereas the general residence requirement for naturalization is 10 years. Until today, and despite the radically changing circumstances, none of this has been modified. The remarkable continuity of Spanish nationality law becomes very clear not only when looking at the (relatively few) reforms that did take place—all of which consolidate rather than change the original normative structure and underlying rationales—but also the various reform proposals that have been rejected over the last decades—many of which would have significantly altered the existing legal framework (see Table 1).

This stability of the Spanish citizenship regime represents a notable exception in the European context. Along with Italy, Spain is the only major destination country that has not reformed its citizenship laws in the face of a fast-changing immigration reality (Huddleston et al., 2015; Pasetti, 2019). Over the last two decades all other major receiving countries in Europe have significantly revised their rules for acquisition and loss of nationality, often in response to perceived challenges posed by growing immigration. Several countries with long-standing *jus sanguinis*-based systems carried out liberalizing steps toward immigrants by introducing citizenship rights based upon birth in the territory. In Germany, for example, a reform in 2000 has lowered residency requirements for naturalization and introduced *jus soli* for children of foreigners with permanent residence rights. Others have headed in the opposite direction. Ireland, for instance, has limited its previously unconditional *jus soli* rules so that now, children born in the country can obtain nationality only on the condition that one of their parents has (lawfully) resided in Ireland for three of the previous four years. Without delving into the political factors and debates that led to these reforms, they provide good examples of how other countries affected by increasing immigration have adapted their citizenship regimes.

So, what lies behind the exceptional stability of Spanish citizenship law? This article aims to provide new answers to this question by shifting the analytical focus to the realm of ideas, which we conceptualize and identify in the form of “frames”, i.e., the interpretive schemes that shape how policymakers perceive, define, and address certain policy issues (Bleich, 2003). Based on a close analysis of the law-making process and of parliamentary debates about actual and potential citizenship reforms between 1978 and 2024, we argue that this puzzling stability can partly be attributed to the widely shared and remarkably stable way in which the country's political elite frames nationality and belonging. Approaching this issue from the theoretical perspective of discursive/constructivist institutionalism allows us to provide an endogenous explanation based on ideas that drive the policymaking process from within. This complements the exogenous accounts developed so far (Finotelli and La Barbera, 2013; Martín-Pérez and Moreno-Fuentes, 2012) that focused on external factors and constraints.

By offering a fuller understanding of the politics of citizenship in Spain, the article makes three distinct yet interconnected contributions to three areas of research. First, to the field of comparative citizenship studies, which has so far focused more on analyzing changes (whether liberalizing or restrictive) in citizenship regimes but has paid little attention to regime stability and continuity. Second, to the research on ideas, which has been more oriented toward explaining ideas as drivers of change, but less prepared to account for them as barriers to change. Finally, to the literature on the citizenship-migration nexus, providing fresh empirical insights into the ambiguous role that ideas about past and present migratory dynamics play in shaping the evolution of citizenship law-making.

The following section provides a review of relevant literature on citizenship politics and policymaking as well as the so-called “migration-citizenship nexus”, thereby both situating our study within this scholarship and providing a theoretical framework for our analysis. Section Methodology briefly presents the methodological approach and its epistemological underpinnings before we present our empirical findings in Section Key ideas behind citizenship law-making in Spain, where we identify three key frames that play a causal role in ensuring law-making stability over time. The discussion and concluding sections summarize the findings and highlight the theoretical and empirical value of the study and analysis.

## Citizenship, migration, and ideas: a theoretical approximation

Both in everyday language and legal jargon, “citizenship” designates an individual's belonging to a particular State (Costa, 2005). In a world without migration this relationship would arguably be of much less concern for national politics: the people who are born in a country would be that country's citizens throughout their entire lives and the same would apply to their children and all subsequent generations. But in a world marked by significant transnational mobility (Castles and Miller, 2009)—much of which preceded the invention of the nation-state (e.g., Manning, 2005)—the relationship between individuals and “their” nation-state is often much more complicated. While human mobility is certainly not the only dynamic that challenges this relationship and thereby troubles the politics of citizenship,^5^ this is one of many ways in which migration and citizenship are intimately connected, as noted by Stasiulis (2008) who was among the first to describe this connection as the “migration-citizenship nexus”. For her, the fact that “migration is a force that splinters, spatially disperses, and complicates citizenship” (Stasiulis, 2008, p. 134) is at the core of this nexus. A related argument was made by Anderson (2019, p. 8), who highlighted that “[t]he instability of the category of “migrant” after all destabilizes the category of “citizen”, like when citizens whose parents had immigrated are referred to as “second generation migrants” and are thereby “migrantizised””. Arguably, something similar might be happening when some foreigners are given privileged access to the citizenship of a certain country—and are treated as (potential) “returnees” rather than immigrants—just because their parents or grandparents had emigrated from that country.

When it comes to concrete policy implications of the “migration-citizenship-nexus”, most authors have come to the conclusion that increasing immigration requires a liberalization or flexibilization of citizenship regimes, in order to accommodate the resulting complexities (ethnic diversity, multiple belongings, etc.) within the normative boundaries of the political community (e.g., Joppke, 2001; Howard, 2009; Domingo and Ortega-Rivera, 2015). It is thereby generally assumed that migration comes before citizenship acquisition. Less often, citizenship—usually in the form of “external” citizenship (i.e., acquired by non-residents)—is regarded not as an outcome but a facilitator, or even driver, of migration, or at least of migratory aspirations and opportunities (e.g., Harpaz, 2019; Džankić and Vink, 2022; Blanchard, 2024). This side of the nexus has been highlighted in relation to “ancestral citizenship” offered to former emigrants' direct descendants, whose subsequent immigration can then be officially framed as a “return” to the (ancestors') country of citizenship, even though in practice it is certainly much more than that (e.g., Blanchard, 2024).

The key function of the citizenship regime is to ensure the state's intergenerational continuity, which involves decisions about the modes of transmission of citizenship (i.e., by descent, via birthplace, and/or through naturalization), the modes and conditions of its loss, and the degree of its exclusivity (Vink and Bauböck, 2013). A government can, for instance, decide to favor the descendants of emigrants over those of immigrants, or to instead privilege the latter, or to facilitate citizenship access for both. It implies choosing the requirements for naturalization, which might (or not) include the obligation to renounce any previous citizenship. Modern nation-states have found different answers to these questions, and despite an apparent trend toward overall convergence (Joppke, 2007; Goodman, 2010), there is still substantial cross-national variation among contemporary citizenship regimes (Vink and Bauböck, 2013).

To explain this variation, scholars have richly drawn from the institutionalist tradition, especially from its rational-choice and historical variants. The contributions by Green-Pedersen and Odmalm (2008) and Brochmann and Seland (2010) can be seen as paradigmatic in this regard. Both studies provide a comparative analysis of the Swedish and Danish naturalization laws; yet whereas the former follows rational-choice institutionalism and hence explains variation based on different coalitional opportunities within the right-wing blocs of the two countries, the latter draws on historical institutionalism and argues that their diverging naturalization regulations can be attributed to dissimilar institutional legacies and to different conceptions of nationhood.

Rational-choice institutionalism explains political action by focusing on institutions conceived as scripts that constrain the behavior of rational and preferences-maximizing actors (Olson, 1965; Shepsle, 2008); thereby focusing on the power and position of political parties (Howard, 2010), coalition opportunities (Bale et al., 2010), and relations with public opinion and other parties (Akkerman, 2015). Abiding by this theoretical approach, liberalizing changes in citizenship regulations are more likely to occur when leftist majorities hold office, and the country lacks a strong right-wing party exploiting anti-immigrant attitudes (Howard, 2010). Historical institutionalism, on the other hand, focuses on the explanatory power of path-dependency (Arthur, 1994; Mahoney, 2000). In the field of citizenship, this approach led to various typologies of national models of citizenship (Jacobs and Rea, 2007) and the idea that nation-states adapt their citizenship regimes to (increasing) immigration based on the political culture and conception of nationhood established during the process of nation-state formation (Brubaker, 1992; Favell, 1998; Koopmans et al., 2012). *Prima facie*, these theoretical approaches seem unable to account for the stability of the Spanish citizenship regime. Both of them, indeed, would arguably lead us to expect a liberalizing change of the framework established in 1982: as reaction to socio-demographic changes in the country following the drastic increase in immigration, according to historical institutionalism; due to comparatively favorable attitudes toward immigration and the absence—at least until 2019—of an anti-immigration party in the national political arena (González Enríquez and Rinken, 2021) following the rational-choice institutionalism.

In the first decade of the twenty-first century an apparent trend of policy convergence inspired new and broader academic thinking that enriched the spectrum of theoretical explanations. As additional factors, scholars have emphasized the homogenizing effect of supranational institutions (Acosta Arcarazo and Geddes, 2013) and the role played by national courts and constitutions in spreading liberal-democratic principles within domestic political arenas (Joppke, 2001, 2010). What also helped contemporary theorisations of citizenship politics to move beyond the juxtaposition of relatively stable national models on one hand, and pressures for convergence on the other (Finotelli and Michalowski, 2012), was the increasing acknowledgment of the role of ideas as an empirical subject to be studied in its own right. Over the last years, several rational-choice and historical institutionalism scholars have headed toward discursive (Schmidt, 2008, 2010) and constructivist institutionalisms (Hay, 2007), focusing on how ideas engage with and are able to shape existing institutions, structures, and political dynamics.

This engagement led to the development of more complex theoretical frameworks to account for the evolution of citizenship regulations over time. Winter (2014) for instance, accounts for the different citizenship policies of Germany and Canada by using the concept of “national trajectories”, which builds on the theoretical backdrop of national models but overcomes the determinism of classic path dependency by emphasizing the influence of national conjunctures and ongoing political imaginaries. As Winter (2014, p. 29) put it, “national trajectories—rather than models—provide a cognitive matrix into which policy changes and their justifications need to be inserted”. In this article, we intend to contribute to this expanding branch of the migration and citizenship literature, by focusing on the case of Spain, and tracing this particular citizenship regime from its beginnings in the early 1980s until today. To do this, we analyze its evolution from an epistemological perspective that has been described as “discursive institutionalism” (Schmidt, 2008, 2010) or “constructivist institutionalism” (Béland and Cox, 2011; Hay, 2007). While there are subtle differences,^6^ both approaches put the focus on ideas as key drivers of policy evolution. In contrast to the above-mentioned older variants of institutionalism, discursive and constructivist institutionalisms understand institutional genesis and transformation as the result of endogenous factors that are sometimes described as “ideas” and conceived as existing structures that influence the actions of political actors but at the same time result from these same actions (Schmidt, 2008). Policy genesis and transformation are not the outcome of external pressures linked to structural conditions but instead result from the interactions between actors and structures, with ideas playing a key role. Various theoretical constructs have been developed to capture ideas as objects of empirical analysis. Sabatier (1987) introduced the concept of “belief system”, Hall (1993) discussed “policy paradigm”, Katzenstein (1996) and Bleich (2003) chose “frames”, and more recently, Schmidt (2008) proposed “background ideational abilities”.

Among the various kinds of “ideas” explored in the literature of discursive and constructivist institutionalisms this study focuses on what has been described as “frames”. While Chong and Druckman (2007) have highlighted the “policy-specific nature” of frames, Bleich (2003) famously described them as sets of cognitive and normative elements that orient an actor within a concrete legislative domain. As cognitive maps, frames contain descriptive and causal assumptions that identify the salient dimensions of an issue. As normative maps, they offer a moral assessment of events, problems, as well as alternative solutions. A causal argument grounded on frames, hence, traces legislative outputs back to the way in which ruling political elites think about—i.e., “frame”—a specific legislative domain. In short, these constructs influence the identification of problems, causal explanations, moral judgments, and proposed solutions, thereby structuring not only political debates but also policy outcomes.

Empirical analyses of the politics of citizenship in Spain have, so far, remained rather distant from the world of ideas. Or, when they have drawn near it, they have loosened their grip on the area of nationality. Finotelli and La Barbera (2013), for example, provide interesting insights into the selection mechanisms deriving from the Spanish “heritage-based” naturalization rules but their study does not delve into an appraisal of the underlying ideational substratum. Gil Araújo (2006) and Zapata-Barrero (2009), on the other hand, do touch on ideas while discussing the existence of a distinctively Spanish “philosophy of integration”, but their analysis covers a much broader spectrum of “integration” policies and says very little about the more specific politics of citizenship and the making of corresponding policies. So far, the most comprehensive analysis of the evolution of the Spanish citizenship regime—including an explanation of its stability—has been provided by Martín-Pérez and Moreno-Fuentes (2012). According to them, the lack of main political parties' interest in reforming this regime, together with a particular “political culture” derived from Spain's colonial past, account for the high degree of stability of citizenship laws in the country. Drawing on insights from rational-choice and historical institutionalism, their analysis provides a sophisticated account of the main political parties' incentive structures and the stringent logic of path-dependency underlying the historical evolution of nationality law. However, they leave ample room for further empirical inquiry into the role of ideas. Our intention is to not only update their examination but also to complement it in this direction, by identifying the sets of ideas that underly the Spanish politics of citizenship and assessing their role in ensuring stability of the resulting legal framework.

## Methodology

Following this theoretical framework, the striking stability of Spanish nationality law can only be fully understood by digging into the ideational substratum underlying the politics of citizenship. Our assumption is that it can at least partly be attributed to the stabilizing role played by certain citizenship frames that are widely shared among Spain's political elite. The empirical quest is thus to show that members of parliament—as key political actors—hold a stable set of ideas when it comes to questions of nationality, that such elements are linked to the institutional stability, and that they are not merely reducible to contextual conditions surrounding the politics of citizenship. In order to do this, we employ a methodological approach that combines process tracing with inductive qualitative content analysis (Hsieh and Shannon, 2005; Winter, 2014). On the one hand, we have thematically coded and analyzed the transcriptions of selected parliamentary debates about actual or proposed citizenship reforms discussed in plenary sessions as well as specialized commissions in both the Congress and the Senate (see Supplementary material). We have analyzed all debates about initiatives with “legislative function” formulated in the Congress both by parties in government and in opposition, excluding those that have “expired” (i.e., have not been debated within the required legislative timeframe) and those “withdrawn” by the proponents themselves. In short, the analysis covers the debates about all legislative initiatives that were subsequently put to a vote. While we included interventions by representatives of all parties represented in the Spanish parliament, particular attention has been paid to the two main political parties that ever since the advent of Spanish democracy have alternated in leading the Spanish government: the center-right *Partido Popular* (PP, Popular Party) and the center-left *Partido Socialista Obrero Español* (PSOE, Spanish Socialist Party). On the other hand, we applied inductive process tracing (Beach and Pedersen, 2013) to disentangle nationality law-making over time and better understand its intimate relationship with the underlying ideational substratum, which we expect to have a significant effect on it. To this end, and in line with the methodological requirements of process tracing applied to ideas (Jacobs, 2015), the analysis of nationality law-making and political discourse is extended over a significant period: from the country's transition to democracy to the present day (1978–2024). This extensive timeframe allows us to verify the stability—and thus the authenticity—of ideas despite changing contextual and political conditions. In other words, it excludes the possibility that the analyzed political discourse, rather than reflecting the genuine frames held by the political elite, reflects a merely strategic and/or rhetorical justification concealing other underlying political interests.

## Key ideas behind citizenship law-making in Spain

In Spain, the notable continuity in citizenship law-making finds a clear reflection—and, we argue, an important part of the explanation—in the concrete and stable way in which the country's political elite has been conceiving and discussing the issue of nationality since the early 1980s. As will be shown in the following, the political discourse we have analyzed shows substantial homogeneity not only over time but also across the political spectrum, whereby ideologically very differently positioned parties' views of citizenship are closely aligned and have remained essentially unaltered throughout almost five decades. More specifically, we identify three constitutive elements of this shared ideational substratum of the Spanish citizenship regime, which help to explain the stability of the latter: The preference for blood-ties over territorial presence, the preferential treatment of emigrants (and their descendants) over immigrants, and a predilection for potential citizens' historical over contemporary connections to Spain. While there certainly is some overlap between these three aspects, they reflect distinct dimensions of the political imaginary of citizenship. The first (and central) dimension is constituted by the well-known dichotomy between *jus sanguinis* (blood-based citizenship attribution) and *jus soli* (territory-based citizenship attribution), which reflects how states navigate competing imperatives of cultural continuity and demographic adaptation. The second dimension more specifically refers to how citizenship laws intersect with migratory dynamics by differentiating between the people who have left (emigrants) and those who have entered (immigrants) the country. The third dimension captures how such differential treatment is justified through temporal rationales—either backward-looking based on historical links or events, or forward-looking based on current demographic developments and needs. In the following we will flesh out these three elements, before reflecting upon their causal role in driving citizenship law-making and their relation to different dimensions of the migration-citizenship nexus.

### Blood over territory

At the core of the ideational substratum underlying (and arguably stabilizing) the Spanish citizenship regime is the legislator's enduring preference for *jus sanguinis* over *jus soli*. This conviction to ensure the continuity of the State primarily through kinship rather than based on who is born and/or resides within the national territory is essentially shared by parties across the political spectrum, as the following quotes indicate:

“Regarding the transcendental issue of the attribution of Spanish nationality by origin, the “ius sanguinis” criterion is maintained, which has been classic in Spain, on historical and political grounds.” (Landelino Lavilla Alsina, UCD—Congress, 3 February 1981, 1211).

“Becoming a national requires a [strong] relationship with the State, which we consider insufficient in the cases of birth on Spanish territory from legally resident [i.e., immigrant] parents.” (Silva Sanchez, CiU ^7^—Congress 20 June 2000, 565). “We believe that it is more important to maintain the concept of *jus sanguinis* in our legal framework [*ordenamiento*] than that of *jus soli*.” (Muñoz Uriol, PP - Congress, 20 May 2002, 15910).

“If we want to open the doors for Spaniards' grandsons and granddaughters to opt for [Spanish nationality], we will have to remove the requirement of being born in Spain because almost all of them, children of emigrants, were born outside our country.” (Villarubia Mediavilla, PSOE—Congress, 20 May 2002, 15915).

According to this widely shared imaginary, the mere fact of being born on Spanish soil is thus not enough to automatically turn a foreigner into a Spaniard, nor should someone's birth outside the Spanish territory restrain the right of Spanish parents to automatically pass their citizenship on to their children. The quotes also suggest that the concern for Spanish ancestry and for the diaspora go hand-in-hand, and that both represent an area of meaning in which transversal political consensus is easily built. A good indication of this is the fact that very similar reforms all aimed at facilitating citizenship access for emigrants' descendants and at reinforcing their ancestral ties with Spain were carried out under both center-left (i.e., law 18/1990; law 29/1995; law 40/2006; law 52/2007) and center-right governments (law 51/1982, law 32/2002) and tended to receive cross-party support.

In contrast, various proposals that would instead have strengthened the principle of *jus soli* and thus the importance of birth and/or continued residence within the country (irrespective of ancestry), ended up being rejected. In relation to this, it is important to highlight the somewhat ambiguous stance that the PSOE has taken over the years in this regard: During the 1990s and early 2000s, when the party was in opposition, it put forward several bill-proposals aimed at strengthening *jus soli* and liberalizing residence-based naturalization (all of which were rejected by the ruling majority); but once the party had won the elections and led the government, its concern for *jus soli* suddenly seemed to have faded, and its reform proposals focused—once again—on the rights and opportunities of emigrants' descendants. Also in this case, not only the parliamentary debates but also the legislative output thus clearly reflects a set of traditional beliefs and values that are widely shared among the country's political elite, including both center-right (PP) and center-left (PSOE) parties, as well as the far right (VOX). In fact, the discourse of VOX is very much in line not only regarding the preference for “blood” ties over territorial presence, but also of emigrants over immigrants, which is a second element that we have identified as key.

### Emigrants over immigrants

Underlying this second core dimension is the widely shared perception that Spanish nationality is primarily a diaspora-related matter. In the political discourse, this goes beyond a mere commemoration of Spain's long and significant emigration history, and instead often takes the shape of a forward-looking political imaginary, in which emigrants and their descendants are considered a “key social capital on which to build the future of the country” (e.g., law 40/2006). As becomes clear during many of the analyzed debates, all political parties share a firm concern for safeguarding emigrants and their descendants' interests, rights, and needs, favoring their return and strengthening their links with the Spanish State; and the area of nationality constitutes the domain in which these concerns are believed to deserve an answer by the national legislator.

“What we intended was to facilitate the reintegration of emigrants […] by exempting them from the requirement of residence in Spain during one year in order to recover their lost Spanish nationality.” (Jordi Solé Tura, PSUC—Congress, 4 May 1982, 13769).

“The Popular Party clearly defines an objective when it wants to modify the Civil Code on issues related to nationality. […] We believe that our essential commitment, which I think is well-founded too, is with emigration and its descendants.” (Muñoz Uriol, PP—Congress, 5 February 2002, 6801).

“The ambition of a country like ours cannot be another than that of guaranteeing the full equality of rights, benefits and opportunities of those living abroad in comparison with those residing within the State.” (Rubial Cachorro, PSOE—Senate, 21 November 2006).

As these quotes indicate, when it comes to diaspora rights, the Popular and Socialist Parties' stances are significantly closer than their very different ideological positions and sharp divisions over most other issues (including immigration) would let us expect. This consensus among otherwise rather disunited parties' views on what could be called the emigration-citizenship nexus also includes various regionalist parties,^8^ which have quite consistently supported reforms aimed at protecting diaspora rights and, in some cases, have even led reform proposals aimed at easing access to nationality for descendants of emigrants'. A representative of the Basque Nationalist Party put it this way:

“[Targeting emigrants' offspring] by way of Immigration Law is alienation, it's like considering grandchildren as strangers to the State, while what we request, and for what we proposed amendments during the whole process, is that they be considered as proper citizens.” (Uría Etxebarría, PNV—Congress, 24 September 2002, 17990).

An additional aspect that helps to explain the prominence of the emigration-citizenship nexus within the political discourse is the institutional architecture that also reflects a particular sensitivity for emigrants' interests and claims. On the one hand, the discursive space given to these interests in the parliamentary arena is quite significant, which becomes very clear when looking at the substantial amount of time regularly dedicated to interventions by emigrant representatives, e.g., in the proceedings of the Law 40/2006 on the Statute of Spanish Citizenship Abroad. On the other hand, those claims also resonate with many parties' organizational structures, which often include a branch specifically dedicated to the diaspora. The proposal for Law 18/1990, for example, was developed by the Secretary of Emigration of the Socialist Party canalizing emigrants' demands.

At the same time, party positions also concur in their apparent lack of ambition to extend (or further restrict) citizenship rights for immigrants living in Spain, even at times when their number and visibility in public life as well as political discourse was growing exponentially. Despite immigration becoming an undeniable reality and structural feature of Spanish society, the political debates about naturalization have remained essentially unchanged, although there is now significantly more variation among the parties in this regard. Until the far-right party VOX had entered the national parliamentary arena in 2019—and with it a much more radical and at times openly xenophobic tone—the spectrum ranged from the most liberal position of *Izquierda Unida* (IU, United Left) to the most restrictive and securitarian one held the Popular Party, and political actors had seldom explicitly referred to immigration or immigrants when debating questions of nationality in parliament. Even the parties that—like the PSOE—traditionally hold favorable stances on immigration and often explicitly promote foreigners' inclusion into Spanish society, only ever openly advocate for also facilitating immigrants' access to citizenship when they are in opposition and there is no parliamentary majority to support such change. Especially during the second half of the 1990, several proposals (by the PSOE or IU) for liberalization of existing naturalization rules have been rejected, and since then, residence-based citizenship acquisition by immigrants in Spain has become more politicized and is nowadays more often explicitly problematized during parliamentary debates, especially since VOX entered the parliamentary arena. It is important to note, however, that also the various proposals to restrict residence-based citizenship acquisition for (especially Moroccan) immigrants—all of which were made by Spain's first far-right party (e.g., 122/000176, 122/000054)—ended up being rejected by a significant majority in parliament (see Table 1). On several other occasions, like the debates around the 2022 Democratic Memory Law, representatives of VOX have instead fallen in line with the accepted canon of ideas regarding the issue of nationality and left aside their otherwise explicit anti-immigrant rhetoric and claims. This canon portrays citizenship not only as a matter of diaspora rights but also as based on the county's historical connections.

### Historical over contemporary ties

A third element that is equally crucial for understanding the overall stability of citizenship in Spain, and that is also characterized by a broad political consensus, is the preferential treatment of two particular groups of foreigners, whose links to the country reach a long way back: citizens of countries that had formerly been Spanish colonies, and Sephardic Jews whose ancestors had been expelled from the Iberian peninsula more than 5 centuries ago. Both groups are described and officially recognized as having a “special link” with Spain and in both cases these ties are based on a historical connection. The privileged citizenship access for citizens of former colonies is thereby often simply assumed to be a legitimate pillar of the country's citizenship regime without explicit mention of the underlying historical relations and the resulting cultural affinities. Notably, the notion of *Hispanidad*, a term rooted in the country's colonial past (Alvarez Rodriguez, 2010) that embraces the idea of enduring cultural proximity based on common religious and linguistic roots between the now independent Latin American countries and Spain, is almost completely absent from the parliamentary debates we analyzed.^9^ The special bond that is supposed to tie all Latin Americans to Spain thus appears like a legacy of the past that is taken for granted within debates around contemporary citizenship.

When it comes to the much more specific group of Sephardic Jews, on the other hand, the political actors' rhetorical strategies are different: identity and cultural elements are more frequently and more specifically spelled out, often with reference to concrete historical events. While there is a certain variation among political parties in terms of which other historical groups should also receive a similar preferential treatment,^10^ they all agree upon the need to not only honor but also make up for the historic injustice suffered by Sephardic Jews during the *Reconquista*. This agreement became most apparent during the debates around Law 12/2015 “on the granting of Spanish citizenship to Sephardic Jews with Spanish origins”, as illustrated by the following quotes by representatives of the PSOE (the main opposition party at the time) and the (then governing) PP:

“We want this law recognizing nationality for Sephardic Jews to be approved to solve a historical injustice with the Spanish Jews. We want it to be approved. We believe it is a just cause.” (Silva Rego, PSOE—Congress, 20 November 2014, 71).

“The meaning of the law is, in some way, to recognize, to honor, to accommodate among us, all those who, in a truly incredible way throughout the centuries, and despite having been so unjustly excluded from living among the Spaniards, have maintained their traditions, have maintained their language, have maintained their cultural roots, have maintained the spiritual bond with Spain.” (Elorriaga Pisarik, PP—Congress, 25 March 2015, 13).

A notable difference between the two parties' stances is that the former puts the historic injustice at the center of how they justify special treatment for this group today, whereas the PP particularly highlights the fact that the people in question have chosen to maintain certain cultural traits and traditions that now substantiate their claim and underpin their right to citizenship access based on a thus not merely historical connection but a cultural proximity that they actively conserved over many generations.

The latter aspect also marks a significant difference between Law 12/2015 and the so-called Historical and Democratic Memory Laws that were enacted in 2007 and 2022, respectively, both under left-wing governments. Neither of the two laws focused primarily on citizenship but both included a relevant “additional disposition” offering not only privileged but in fact almost unconditional citizenship access for the children and grandchildren of Spanish exiles who had left the country during the civil war and subsequent dictatorship. The inclination—especially among the political left—to use nationality as a kind of “compensation” (*recompensa*) for historical wrongs, including the injustices suffered by parts of the population under the Franco-regime, was not new but had already appeared in debates about earlier reform proposals (including the one that was ultimately approved in Law 40/2006). And while both the 2007 and 2022 laws were highly controversial and thus hotly debated in parliament, these political contestations were not about citizenship. In fact, during the various debates^11^ about the PSOE's proposal (121/000064) that ultimately resulted in the 2022 Democratic Memory Law few parliamentarians even mentioned the change in terms of citizenship access—as one among many measures to be put in place—and none of them questioned or substantially contested this specific change. After all, the use of citizenship as a form of restitution does not constitute a break with the existing citizenship frame but instead merely serves as an additional argument for extending citizenship rights based on both ancestry (“blood ties”) and emigration (in this case, involuntary exile). Like the very similar changes introduced by the 2007 Historical Memory Law, the reform was thus very much in line with the traditional ideational substratum.

Interestingly, also representatives of VOX have at times emphasized a historical dimension of belonging to the nation, but in stark contrast to the previous examples have turned it into an argument for restricting citizenship rights and excluding who they perceive as unwanted immigrants from the political community:

“Well, for us, being Spanish is much more than having a piece of paper. [...] We think that the nation is not a paper with a stamp, we think that the nation is a moral community, it is a historical project [...], it is a historical enterprise and, therefore, it cannot be open to just anyone.” (Contreras Peláez, VOX—Congress, 15 February 2022, 18).

Overall, the idea of having (or not) a “special link with Spain” (*especial vinculación con España*) emerges as a key concept underlying the Spanish citizenship frame. It has been transversally mobilized by different parties to justify not only privileges but also exclusion from access to nationality, whereby in most cases the underlying links had some historical connotation.

One—in the Spanish context rather uncommon—attempt of making citizenship acquisition somewhat more dependent on potential citizens' individual and *contemporary* ties with Spain was the introduction^12^ of compulsory language and civic knowledge tests for immigrants applying for naturalization, which replaced the previous system based on individual interviews conducted by local judges. Whereas in countries like France, Germany and the UK, the introduction of civic integration tests generated intense public and political debates (Carrera, 2006; Mouritsen, 2012), this has not been the case in Spain, where the change passed almost unnoticed by the broader public^13^ and without any thorough discussion of the underlying meaning and possible implications for the people wanting to become Spanish nationals. This development has not been accompanied by a de-ethnicization of citizenship, as suggested by Joppke (2007), nor was it intended to hide the clearly ethno-centric conception of belonging, as the critics of the “liberal-convergence” thesis argued (e.g., Goodman, 2012). Instead, the reform has been justified with the need to streamline the administrative procedure and ensure consistency in the face of a significant backlog of naturalization applications. While this change happened years before VOX had entered the parliamentary stage, the logic of obligatory civic integration tests is very much in line with this party's rhetoric, as the following quote from a debate in 2022 (about proposal 122/000176) makes clear:

“It is always the responsibility of those who come from abroad to make the effort to adapt to the morals and customs of the country, and only those who are willing to embrace the productivity, culture, history and language of Spain deserve our Spanish nationality, which is a high honor.” (Contreras Peláez, VOX—Congress, 15 February 2022, 19).

When it comes to the offspring of Spanish emigrants, in contrast, representatives of all political parties seem to simply assume this same willingness to exist automatically—like in the case of the descendants of Spanish exiles who are offered Spanish citizenship without having to comply with any integration requirements, even though technically also they will “come from abroad” if they eventually decide to “return” to their ancestors' homeland.

## Discussion

As we have shown, the striking continuity in Spanish nationality law-making is underpinned by a dominant citizenship frame that has been maintained over time and shared across political parties, focusing on blood over territory, emigrants over immigrants, and historical over contemporary ties. The resulting stability of the legal framework signifies more than a mere absence of change. In fact, the original nationality regime established back in 1982 has been renewed multiple times over the period we analyzed, but always in the same direction: prioritizing the descendants of emigrants and honoring certain groups' historical ties while leaving aside the claims for belonging made by other collectives, including non-Hispanic immigrants who have been living within the country for many years. The underlying political dynamic is not merely a relic of the past nor a reaffirmation of Spain's historical identity as a country of emigration. It also results from a particular and widely shared conceptualization of citizenship: while Spain became one of Europe's main immigration countries, its political elite actively decided to continue to view citizenship as primarily related to Spaniards abroad.

This suggests that the stability in nationality law-making is not simply due to path-dependency, as emphasized by Martín-Pérez and Moreno-Fuentes (2012), but also reflects an “ideational hegemony of an entire policy sector,” as Schmidt (2011, p. 100) would call it, or, in other words, a dominant citizenship frame shared across political parties. This dominant frame has found a fertile ground on which to develop in both major parties' pragmatism and normative constrictions. A good example of the former is the Socialist Party's two-faced attitude regarding a strengthening of *jus soli*, which it only supported when in opposition while turning back to the diaspora when holding office. Martín-Pérez and Moreno-Fuentes (2012) interpreted this behavior as an attempt to prevent the politicization of nationality matters by keeping them separate from the issue of immigration. Seen from the perspective of constructivist/discursive institutionalism, the party's seemingly ambiguous stance and action can also be seen as the result of certain citizenship frames melting into strategic reasoning in the face of existing political contingences. That said, it is also revealing that all the reforms brought about by the PSOE when holding office, ultimately focused on the diaspora. This includes the so-called “Historical Memory Law” of 2007 as well as the “Democratic Memory Law” of 2022, both of which extended citizenship rights to direct descendants (children and grandchildren) of Spanish exiles who left Spain during the Civil War and subsequent dictatorship. The fact that both of these reforms were very much in line with the traditional ideational substratum—linked to emigration, ancestry, and history—explains why there was hardly any political debate about them in parliament, while the broader laws that introduced them were highly controversial and indeed very intensely debated.

Arguably, this dominant citizenship frame has also been favored by the unusual normative venue in which the regulation of Spanish nationality found its place. The fact that nationality rules are set out in the civil code, rather than a dedicated law, has represented a further impediment for a change of perspective that would have linked citizenship more explicitly to the immigration phenomenon, and instead facilitated an often rather procedural understanding of citizenship acquisition. The shift from individual interviews to standardized language and civic naturalization tests (in 2015), for example, could have been officially framed as an attempt to give more weight to the actual (rather than historical) ties that immigrants applying for naturalization have with Spain. Instead, and in stark contrast to many other countries where similar reforms triggered intense discussions over the underlying meaning and unequal effects of this measure, it was approved without much debate and justified simply in terms of an administrative need to standardize and thereby streamline the naturalization procedure itself.

The nexus between citizenship and immigration has—as we already mentioned—seldom been openly recognized in political debates about Spanish citizenship law and potential reforms thereof. During one of the initial parliamentary debates about the law that gave birth to the Spanish citizenship regime in 1982, the then minister of justice described nationality as “the personal element that determines the Spanish State”, highlighting that “in this sense, a law on nationality has the significance of a law on borders, in that it aims to define the personal boundaries of the state” (Cabanillas Gallas, PP—Congress 27 Abril 1982, 13660). At least indirectly, this does link the debate about citizenship to immigration and border control, and thereby stands in contrast to more frequent discursive attempts to keep the issue of immigration outside of these debates. For example, when discussing a proposed (and ultimately rejected) citizenship reform that would have strengthened *jus soli* and liberalized naturalization rules for immigrants, a representative of the (then governing) PP argued that:

“One thing is the phenomenon of immigration, and another thing is the debate on nationality; they are two concepts that do not necessarily have to be linked. [...] To ground a reform of nationality on the phenomenon of migration does not seem right to us.” (Jordano Salinas, PP—Congress, 14 December 1999, 15073).

Only in very few instances have parliamentarians alluded to the potential effects that citizenship reforms might have in terms of future immigration dynamics; and those who did, made sure to frame these dynamics as a “return” to an ancestral homeland. In his legal analysis of the Law 36/2002, Carrascosa González (2002) noted that the new regulation “logically promotes the possible return to Spain of […] the children of Spanish emigrants [living] abroad” whereas it did not extend the same opportunity to emigrants' grandchildren, a fact that the author interpreted in the following way:

“Undoubtedly, the intention of the legislator [PP] has been to avoid an ‘avalanche' of grandchildren of Spanish emigrants living abroad who are fleeing their host countries due to the economic crisis ravaging those countries and who now want to return and settle in Spain. For this reason, their access to Spanish nationality is more restricted than that envisaged for the children of Spanish emigrants abroad.”

This interpretation would suggest that Spanish lawmakers are indeed aware that citizenship must be imagined and legally framed in a way that does take into account not only past but also present and future migrations affecting the country. Seen from this perspective, the fact that both the Historical and Democratic Memory Laws—which did extend citizenship access to emigrants' grandchildren—established a relatively tight timeframe (of initially 2 years, extended by 1 year) for them to apply for citizenship “restitution”, could be argued to reflect the same concerns. The framing of ancestral citizenship as a necessary means to facilitate—although within certain limits—the so-called “return” of people who have never lived (or even been) in Spain but descend from a Spanish emigrant reflects all three of the key elements that—we argue—help stabilize the Spanish Citizenship frame. And it also constitutes an important feature of the migration-citizenship nexus that is only beginning to receive scholarly attention (see e.g., Blanchard and Lemarche, 2023; Blanchard, 2024) that so far, however, tends to privilege the perspective of the people acquiring a second citizenship, rather than those involved in making the corresponding laws.

## Conclusion

As thoroughly demonstrated (Brubaker, 1992; Favell, 1998; Castles and Miller, 2009), changing migration dynamics normally lead receiving countries to reform the system of rules regulating the acquisition and loss of nationality. More specifically, Džankić and Vink (2022, p. 357) have argued that these rules tend to be affected not only “by modern migratory flows”, but also by the “political and economic contestation over migration—be it immigration or emigration” (p. 359). In the case of Spain, however, neither the sharply increasing inflow of migrants (particularly at the beginning of the 2000s), nor the now notably increasing politicization of the topic (triggered by the appearance of VOX) had any significant impact on citizenship legislation. While VOX has certainly added a new and often quite openly xenophobic tone to the debate and contributed to a certain polarization of the political discourse regarding not only immigration but also citizenship, this has not—at least to date—significantly changed the ideational substratum of the Spanish citizenship regime.

The present article has shown that this puzzling stability of Spanish citizenship law can only be fully explained by looking more closely at how the country's ruling political elite thinks about citizenship itself. The Spanish citizenship frame has outlived extraordinary demographic transformations, changing governing coalitions, as well as the surge of the country's first far right and explicitly anti-immigrant party. Access to citizenship continues to be framed in terms of bloodlines and as a means to reinforce the historical links that tie certain groups—including the descendants of former emigrants and immigrants from former colonies—to the Spanish homeland. This is often combined with an instrumental view on the diaspora—as a driver of economic growth and development—and sometimes justified as a form of restitution for injustices suffered in a more or less distant past. This framing stretches across different and ideologically opposed political parties and, for more than 40 years, has remained essentially unchanged. The presence of such dominant citizenship frame cutting across the parliamentary arena has made it easy to find political consensus in nationality matters. A transversal political agreement regarding the way in which Spanish nationality is to be conceived has secured law-making continuity over time, preventing more substantial political discussions about nationality as part of the immigration debate, which did intensify especially over the last decade. Our analysis suggests that these underlying ideas are not mere rhetorical devices strategically used by political actors to hide otherwise different interests. Instead, the steadiness of this frame within the politics of citizenship suggests a causal role of ideas in shaping the evolution of the Spanish citizenship regime.

The article contributes to three distinct areas of research: Firstly, to comparative citizenship studies, which has so far been more concerned with explaining policy change—whether in terms of a liberalization or tightening of citizenship regulations—rather than policy stability. In this sense, our analysis of the Spanish case enriches the empirical variety and depth of this rapidly expanding field of study by highlighting additional factors shaping the evolution of citizenship over time. Our endogenous account based on ideas usefully complements previous explanations provided by scholars focusing more on the external factors, such as inherited rules, political culture and parties' strategic interests (see Finotelli and La Barbera, 2013; Martín-Pérez and Moreno-Fuentes, 2012). All of these factors certainly also have an influence on legislative action, but they represent only part of the story. As our analysis shows, nationality law-making is also very much about ideas: the beliefs, expectations, and assumptions held by political actors play a crucial role in determining the substance and character of the relevant laws and their evolution or stability over time. In this sense, and secondly, the article also contributes to the broader debate on the role of ideas in public policymaking, where in trying to overcome the limitations of classic neo-institutionalist approaches scholars have turned to ideas mainly in order to explain change (see, for instance, Boswell and Hampshire, 2017 or Carstensen and Schmidt, 2017). Much less has been said about how ideas and their interplay with political decision-making can also prevent such change. This article thus constitutes an important step toward a better understanding of the features and workings of ideas as drivers of legal and institutional stability. And finally, it also enriches the more recent but growing body of literature on the migration-citizenship nexus by shedding new light on the intricate relationship between evolving citizenship frameworks and the migratory dynamics that they seek to respond to (or not). While much of this literature has so far predominantly focused on the perspective, decisions, and practices of individuals acquiring citizenship, this article shifts the focus to the people responsible for making the corresponding rules. Our analysis shows that the way in which Spanish lawmakers have been framing and maintaining this highly differential citizenship regime ultimately reflects an enduring lack of recognition of an *im*migration-citizenship nexus among the political elite and their simultaneous overemphasis on an essentially backward-looking *e*migration-citizenship nexus. The article thereby highlights the value of tracing this nexus also at the level of ideas and within political imaginaries about citizenship, migration, and the nation.

## Data Availability

The original contributions presented in the study are included in the article/Supplementary material, further inquiries can be directed to the corresponding author.

## Footnotes

^1^Italy, for example, has experienced a similar change, but over a slightly longer period and at a relatively smaller scale.

^2^Law 36/2002, Law 40/2006, Law 52/2007, Law 20/2011.

^3^For other conceptualizations see, for instance, Bauböck (2008), Kymlicka and Norman (2000), and Shachar et al. (2017).

^4^In this sense, Spain is no exception within Europe, and it is important to note that Law 51/1982 also already included elements of *jus soli*, by granting citizenship to immigrant children born in Spain if at least one parent had also been born in Spain, and to those who would otherwise be stateless.

^5^The same can be said about the shifting of (or contestations over) international borders, the presence of minority groups within them, or other historical legacies of colonialism.

^6^The main difference between constructivist and discursive institutionalisms regards their different ontological and epistemological stands on discourse. While constructivist institutionalists generally understand “discourse” as the mere empirical substratum through which they identify ideas, discursive institutionalism conceives it “as a more generic term that encompasses not only the substantive content of ideas but also the interactive processes by which ideas are conveyed” (Schmidt, 2008, p. 305), acknowledging to it a specific causal role.

^7^Convergència i Unió (CiU, Convergence and Union) is a center-right Catalan regionalist party.

^8^This is particularly the case of the *Partido Nacionalista Vasco* (PNV, Basque Nationalist Party), *Bloque Nacionalista Galego* (BNG, Galician Nationalist Bloc, BNG) and *Esquerra Republicana de Catalunya* (ERC, Republican Left of Catalonia).

^9^Until 2019, the only reference we found had been made by Cabanilla Gallas (PP) during the debate about the initial reform of the Civil Code back in 1982. After 2019 and the appearance of VOX, the term has become more frequently used in other parliamentary debates (particularly including those regarding VOX-led proposals to reduce immigration and/or immigrants' rights) but not when citizenship itself was the issue under debate.

^10^Apart from the recognition of Sephardic Jews, which is agreed by all factions, PSOE and IU have often stood up for Morisco and Saharan populations to receive similar rights.

^11^Three in Congress, two in the Senate; all between October 2021 and October 2022.

^12^In 2015, through Final Provision seven of the Law 19/2015.

^13^None of the opposition parties brought the issue to the attention of the public when the PP tried to introduce this provision through an amendment to Law 12/2015; and when few months later it was eventually approved as part of the above-mentioned reform of the civic registry (Law 19/2015) they voiced only very cautious criticism.
