# Supplementary material for: Looking back and abroad while (not) moving forward. Migration, ideas and the stability of citizenship in Spain
Source: Front Sociol. 2025 Jul 3;10:1570110. doi: 10.3389/fsoc.2025.1570110 (PMC12267229; doi:10.3389/fsoc.2025.1570110)
Supplement: Supplementary file 3 [file Supplementary_file_3.docx]

**Main coding dimensions for assessing Spanish ruling political elite’s discourse on citizenship**

| **Citizenship Features** | **Codes** | | **Label** | **Description** |
| --- | --- | --- | --- | --- |
| *Ethnic vs Civic conception of citizenship* | Blood-based | | BLO | Intergenerational continuity is ensured through jus sanguinis, privileging an “ethnic” conception of citizenship. |
|  | Territorial-based | | TER | Intergenerational continuity is ensured through jus soli, privileging an “civic” conception of citizenship. |
|  |  | Inclusive | TE_IN | Civic conception is broadly understood as positively associated with value pluralism. |
|  |  | Exclusive | TE_EX | Civic conception is narrowly restricted to liberalism and western values. |
| *Moral grounds of citizenship* | Justice and Equality | | EQU | The idea of citizenship is positively associated with and/or grounded on the principle of social justice and the equality of all human beings. |
|  | Liberty and Individual freedom | | IND | The idea of citizenship is positively associated with and/or grounded on the autonomy of the person and the principle of individual freedom. |
|  | Other moral values/principles | | MOR_OT | The idea of citizenship is positively associated with and/or grounded on the other moral values/principles (in case, specify the value/principle). |
| *Rationale underlying citizenship as formal legal status* | Deserved | | DES | Appeal to the principle of equality between citizens and non-citizens and/or to the right of the latter to be acknowledged as full member of the host society. |
|  | To be deserved | | BED | Nationality is something to be deserved: non-citizens must demonstrate the willingness and/or to be worth to be recognized formal members of the host society. |
| *Bonds with national identity* | Internal dimension | Castillian | CAS | Castillian is acknowledged as the overreaching Spanish identity. |
|  |  | Multin. | MUL | Spanish identity is considered inherently multinational. |
|  | External dimension | Global | NIE_GL | Spanish national identity is positively associated to foreign identities. |
|  |  | Hispanic | NIE_HI | Spanish national identity is positively associated to Hispanic identity (Latin American countries). |
|  |  | Other | NIE_OT | Spanish national identity is positively associated to another foreign regional identity (in case, specify the country). |
| *Historical memories* | Colonialism | Neutral | COL= | Citizenship is debated in relation to the Spanish colonial past. |
|  |  | Positive | COL+ | Citizenship is positively related to the Spanish colonial past. |
|  |  | Negative | COL- | Citizenship is negatively related to the Spanish colonial past (e.g. logic of “compensation”). |
|  | Francoist Spain | Neutral | FRA= | Citizenship is debated in relation to the Francoist period. |
|  |  | Positive | FRA+ | Citizenship is positively related to the to the Francoist period. |
|  |  | Negative | FRA- | Citizenship is negatively related to the to the Francoist period (e.g. logic of “compensation”). |
|  | Other | | HIS_OT | Citizenship is debated in relation to another important historical period (in case, specify the period). |
| *Links with religious faiths* | Christianity  (Catholicism) | Neutral | CHR= | Citizenship matters are related to the Christian religion (Catholicism). |
|  |  | Positive | CHR+ | Citizenship matters are discussed and positively related to the Christian religion (Catholicism). |
|  |  | Negative | CHR- | Citizenship matters are discussed and negatively related to the Christian religion (Catholicism). |
|  | Islam | Nueutral | ISL= | Citizenship matters are related to the Islamic religion. |
|  |  | Positive | ISL+ | Citizenship matters are discussed and positively related to the Islamic religion. |
|  |  | Negative | ISL- | Citizenship matters are discussed and negatively related to the Islamic religion. |
|  | Judaism | Neutral | JUD= | Citizenship matters are related to the Jewish religion. |
|  |  | Positive | JUD+ | Citizenship matters are discussed and positively related to the Jewish religion. |
|  |  | Negative | JUD- | Citizenship matters are discussed and negatively related to the Jewish religion. |
|  | Other | Neutral | REL_OT= | Citizenship matters are related to another religion |
|  |  | Positive | REL_OT= | Citizenship matters are discussed and positively related to another religion. |
|  |  | Negative | REL_OT= | Citizenship matters are discussed and negatively related to another religion. |
| *Relation with international migration (a): immigrants* | General attitude towards immigration and integration | Neutral | ATI= | International immigration and immigrants’ integration are “objectively” understood as relevant political issues and challenges. |
|  |  | Positive | ATI+ | International immigration and immigrants’ integration are opportunities for the growth and the progress of the host society. |
|  |  | Negative | ATI- | International immigration and immigrants’ integration are obstacles to the growth and the progress of the host society. |
|  | Target group | Global | ITG_GL | Mention to international immigration and immigrants’ integration refers to the broad set of immigrants considered as a whole. |
|  |  | Hispanic | ITG_HI | Mention to international immigration and immigrants’ integration refers particularly to Latin-American immigrants. |
|  |  | Other | ITG_OT | Mention to international immigration and immigrants’ integration refers particularly to immigrants coming from another specific country (in case, specify the country). |
|  | Nationality and immigrants’ integration | End | END | Nationality acquisition is considered the final stage of the integration process. |
|  |  | Mean | MEAN | Nationality acquisition is considered a mean for reaching full integration. |
| *Relation with international migration (b): emigrants* | General attitude towards Spanish emigration and diaspora | Neutral | ATE= | Spanish emigration and diaspora are “objectively” understood as a relevant political issues and challenges. |
|  |  | Positive | ATE+ | Mentions to the importance of favoring and safeguarding Spanish emigrants while strengthen the ties with diaspora communities abroad. |
|  |  | Negative | ATE- | Emigrants needs and the strengthening of the ties with the diaspora communities are not worth of political concern. |
|  | Target group | Global | ETG_GL | Mention to emigration and diaspora refers to the broad set of emigrant, regardless of the countries of destination. |
|  |  | Hispanic | ETG_HI | Mention to emigration and diaspora refers particularly to people living in Latin-American countries. |
|  |  | Other | ETG_OT | Mention to emigration and diaspora refers particularly to people living in another specific country (in case, specify the country) |
